# Lead and copper removal from sterile dumps by phytoremediation with *Robinia pseudoacacia*

**DOI:** 10.1038/s41598-024-60412-z

**Published:** 2024-04-29

**Authors:** Adriana Mihaela Chirilă Băbău, Valer Micle, Gianina Elena Damian, Ioana Monica Sur

**Affiliations:** 1 Department of Environment Engineering and Entrepreneurship of Sustainable Development, Faculty of Materials and Environmental Engineering, Technical University of Cluj-Napoca, 103-105 Muncii Avenue, 400 641 Cluj-Napoca, Romania; 2 Department of Cadastre, Civil and Environmental Engineering, “1 Decembrie 1918” University of Alba Iulia, Alba Iulia, Romania

**Keywords:** Pollution remediation, Environmental monitoring

## Abstract

In Romania, huge quantities of gangue material from the mining activity practiced in the past were improperly stored and led to the pollution of the environment. Thus, this work is framed to manage the sterile dump of the “Radeș” mine (Alba, Romania) through a 12-week phytoremediation process. The efficient use of *Robinia pseudoacacia* was studied through the implementation, at the laboratory level, of a phytoremediation experiment based on various variants prepared by mixtures of gangue material, uncontaminated soil, and dehydrated sludge. The prepared variants, all planted with *R. pseudoacacia*, were watered with tap water, potassium monobasic phosphate, and enzyme solution. The bioconcentration and translocation factors for lead showed values ˂ 1, which indicates a potential presence of an exclusion system for Pb or a reduced Pb bioavailability since the *R. pseudoacacia* accumulates high concentrations of metals absorbed on and inside the roots. For copper, both factors had values > 1 indicating the suitability of *R. pseudoacacia* to readily translocate copper into the epigean organs. In the investigated experimental conditions, the highest efficiency in the removal of copper (93.0%) and lead (66.4%) by plants was obtained when gangue material was not mixed with other materials and wetted with enzymatic solution.

## Introduction

Human, urbanization, and industrial activities have led over time to significant changes in the environmental components (water, air, and soil) by introducing numerous polluting substances into the environment including oxides of sulphur, nitrogen, carbon, organic compounds, metals, and compounds of metals, chlorine, fluorine, and cyanides.

Of these, heavy metals are generally associated with the conduct of activities in the mining and metallurgical industries. Heavy metals such as copper, cadmium, lead, zinc, mercury, and arsenic, contained in the residues from mining and metallurgical operations are often dispersed by wind and/or water after their deposition. Unlike other pollutants, heavy metals are highly resistant to either biologically or chemically induced degradation and present a significant risk both for the environment and for human health due to the bioaccumulative character and teratogenic, carcinogenic, and mutagenic effects on living organisms upon exposure^1–3^.

A variety of remediation methods have been developed over the last decades to reclaim heavy metal-contaminated sites^4^. An environmentally friendly depollution technology that attracts attention through its elegance, and scientific and commercial value is phytoremediation. Up to now, significant achievements have been attained during soil and water heavy metal remediation using phytoremediation^5,6^. Comprehensive information regarding the techniques, characteristics and types of plants, factors affecting the process, mechanisms of heavy metal hyperaccumulation, advantages and limitations, and aids or stimulators that improve phytoremediation is available^7–14^.

In an attempt to identify the most suitable and appropriate plant for phytoremedia-tion, different researchers have applied and tested the phytoremediation technology on soils contaminated with heavy metals using various plant species^15–18^. Thanks to their specific characteristics (frost and drought-resistant, grows well in degraded habitats, tolerance to heavy metals) the *Robinia pseudoacacia (black locust)* was widely investigated for phytoremediation of heavy metal polluted soils^13–15,18,19^. The results indicated that the leaves of *Robinia pseudoacacia* can accumulate important quantities of Pb, Zn, and Cu, the quantity increasing with their concentration in the contaminated soil. For example, the accumulation of zinc (19.0 mg kg^−1^), lead (30.7 mg kg^−1^), and copper (17.2 mg kg^−1^) in *Robinia pseudoacacia* leaves from soil contaminated with 38.2, 77.4, and 101.3 mg kg^−1^ of Pb, Zn, and Cu was reported to be about 1.37, 1.38, and 2.15 times higher as compared to the control, respectively^19^.

Currently, phytoremediation technology efforts have focused on using plants to accelerate the reduction or mitigation effects of heavy metals from solid wastes in mining areas. Thus, *Solanum viarum Dunal*, *Quercus* spp. and *Salix* spp. species were successfully used for phytoremediation of mining tailings contaminated with Cu, Pb, Zn, Cr, and Cd^20–22^. Also, *Robinia pseudoacacia*, *Pinus elliottii Engelmann* and *Populus tomentosa Carr*. can reduce the impact of heavy metals in high-density sludge sediment of copper mines^23^. However, the growth environment in mining areas characterized by a large heterogeneity of the substrate has a great influence on the effect of phytoremediation and there are basic science knowledge gaps related to the potential of plants in phytoremediation of mining dumps. For the growth of most plant species, sterile dumps are limiting environments due to unfavorable conditions related to the properties of the substrates like low levels of nutrients (K, N, P) and organic matter, significant amounts of heavy metals, and poor structure^24^. Therefore, the improvement of plant growth conditions by amendments and by treatments (fertilizers) to create larger nutrient concentration, larger water retention capacity, and an improved macrobiotic community in the root areas have been listed among complementary remediation strategies to phytoremediation^25^. Furthermore, mixing the sterile material with unpolluted soil, compost, or organic wastes like dewatered sludge resulting from sewage treatment plants to positively influence plant growth, achieve better plant nutrient statuses, and modify the physical–chemical properties of mine substrates have also been reported in studies of soil and tailing dams^24–26^.

Although the potential of some plant species for phytoremediation of solid wastes in mining areas (e.g. mining tailings, sludge sediments) was explored^20–23^, in the particular case of copper and lead-contaminated mining dumps, the number of studies reporting the phytoremediation properties of plant species in general^24,27^, and of *Robinia pseudoacacia* in particular, is very scarce. These particular metals were chosen to be studied because of their high occurrence in sterile dumps derived from grounding and flotation processes of polymetallic sulphide ores.

Therefore, the present work aims to determine the efficiency of using *Robinia pseudoacacia* in the process of phytoremediation of the sterile dump “Radeș” from Almașu Mare Commune, Alba County, Romania, by implementing, at the laboratory level, a phytoremediation experiment based on various variants prepared by mixtures of gangue material, uncontaminated soil, and dehydrated sludge. The ability of the *Robinia pseudoacacia* plant to remediate the investigated sterile dump through phytoextraction/phytostabilization has been studied alongside the effect of irrigation with potassium monobasic phosphate (KH_2_PO_4_—99.5%) and enzymatic solution during *Robinia pseudoacacia* plants growth on the prepared mixtures. These treatments were applied to ensure the *Robinia pseudoacacia* plants growth conditions and availability of nutrients for plant. Potassium monobasic phosphate was used as a P and K source for plant growth and development while the enzymatic solution was applied to dispose of the mineral ions and facilitate their absorption by the plant. This enzymatic solution contains cellulases along with proteases, lipases, and glycolytic enzymes that are essential for converting cellulose into glucose being the key players in the decomposition of soil organic matter to convert it into plant-available nutrients. Lipases enzymes are vital for assessing soil decontamination and play a crucial role in detoxifying harmful substances^28^. Some aspects regarding the tolerance mechanism of plants when exposed to stress conditions related to the presence of huge quantities of heavy metals, the effect of antioxidant enzymes in phytoremediation, and future perspectives were also discussed. The significance of this work is mostly related to applied science gaps in the field of the application of *Robinia pseudoacacia* for the phytoremediation of copper and lead-contaminated mining dumps.

## Results

### Concentrations of lead and copper present in the prepared experimental variants and plant parts of black locust

**Figure 1:**
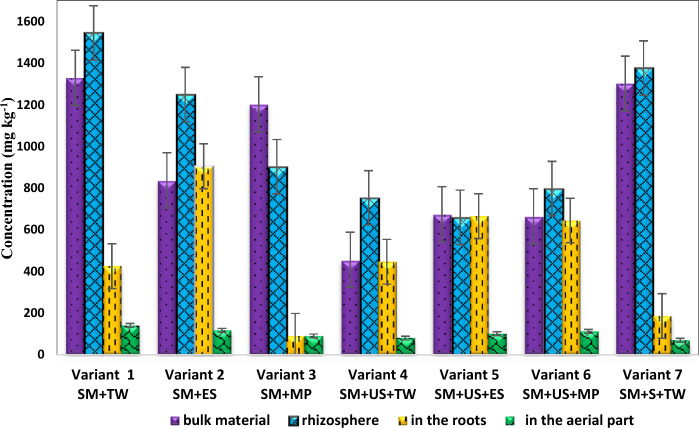
The average concentration of lead present in the samples taken from the bulk material, rhizosphere , and aerial parts of *black locust* specific to the prepared variants. *SM* sterile material, *TW* tap water, *ES* enzyme solution, *MP* solution based on KH_2_PO_4_, *US* unpolluted soil, *S* sludge.

**Figure 2:**
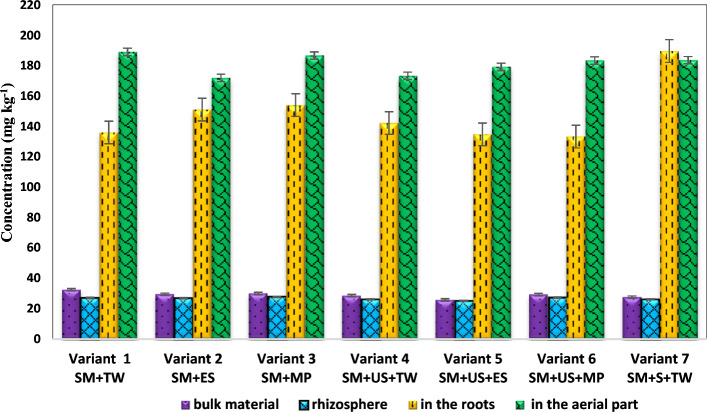
The average concentration of copper present in the samples taken from the bulk material, rhizosphere, and aerial parts of *black locust* specific to the prepared variants. *SM* sterile material, *TW* tap water, *ES* enzyme solution, *MP* solution based on KH_2_PO_4_, *US* unpolluted soil, *S* sludge.

Figures 1 and 2 show the concentrations of lead and copper, respectively, found in the samples taken from the bulk material, from the rhizosphere of *black locust*, from the roots, and their aerial part (stems + leaves) after the phytoremediation process in the case of every prepared experimental variant.

As can be seen in Fig. 1, the lead concentration in the material from the rhizosphere of *black locust* ranges depending on the prepared experimental variant. Variant 0 (reference soil) did not record traces of heavy metals (data not shown). The experimental variant which contained 100% sterile material (variant 1) recorded a lead concentration of 1543.7 mg kg^−1^. 1247.7 mg kg^−1^ of lead was recorded in the experimental variant 2 composed of sterile material irrigated with enzyme solution. Experimental variant 3 composed also of 100% sterile material but irrigated with KH_2_PO_4_ (monobasic potassium phosphate with molecular weight 136.084 g mol^−1^) solution recorded a value of 901.7 mg kg^−1^ lead. In the case of experimental variants 4, 5, and 6, in which the sterile material was mixed with unpolluted soil, the values of lead concentrations in the rhizosphere of *black locust* were in the range of 659.0–796.6 mg kg^−1^. The experimental variant 7 in which sterile material was mixed with sludge, instead of unpolluted soil (variants 4, 5, and 6) recorded a value of lead concentrations in the rhizosphere of *black locust* equal to 1375 mg kg^−1^.

At the *R. pseudoacacia* roots level, the average concentrations of lead varied between 90.4 and 904.0 mg kg^−1^. In the experimental variant 2 composed of sterile material irrigated with enzyme solution a value of 904.0 mg kg^−1^ of lead was measured. In the case of experimental variant 3, also composed of 100% sterile material but irrigated with KH_2_PO_4_ solution a concentration of 90.4 mg kg^−1^ lead was obtained. As compared to variant 3, the roots of the *black locust* developed in variant 6 accumulated 643.6 mg kg^−1^ of lead. The only difference in the content of variant 3 compared to variant 6 was that variant 6 contained 50% of unpolluted soil alongside sterile material.

As compared with the lead content in the rhizosphere and in the roots of *black locust*, in the dry biomass of the aerial parts of the *R. pseudoacacia*, the concentration varied between 69 and 140.96 mg kg^−1^.

In the case of experimental variant 1 the lead concentration was 140.96 mg kg^−1^, while in the case of experimental variant 2 a value of 117.02 mg kg^−1^ was recorded. Experimental variant 5 showed a lead concentration in the aerial parts of 101.0 mg kg^−1^, and in the case of experimental variant 6, the lead content was 111 mg kg^−1^. In the experimental variants 3, 4, and 7, the lead concentration in the aerial parts was in the range of 69–90 mg kg^−1^.

In the case of copper, the cumulative effect of concentrations was the opposite. As can be seen in Fig. 2, the highest concentrations were found in the aerial part of the *black locust* (stems + leaves) and in the roots. In the roots, the copper concentration accumulated ranges from 133 to 190 mg kg^−1^. In the aerial parts of *black locust*, the translocated concentration was situated within 172–189 mg kg^−1^, while in the rhizosphere of *black locust,* the concentrations range from 25 to 27 mg kg^−1^. The copper concentration in the material from the rhizosphere of *black locust* recorded similar values to the average values of Cu concentrations from our experiment preparations, these being located around values of 27 mg kg^−1^ in the event of all variants.

At the end of the phytoremediation experiment the copper concentration from the prepared experimental variants (bulk material) recorded values lower than 33 mg kg^−1^. The lead concentrations found in the prepared experimental variants vary between 455.84 and 1328.0 mg kg^−1^.

The value reported after the measurements represents the arithmetic mean of the experimental results. Following the calculations regarding the mean square deviation, a measurement uncertainty of min 1% and max 5% of the average value of the determined metal concentrations was identified.

### Bioconcentration and translocation factors of lead and copper in *Robinia pseudoacacia*

**Figure 3:**
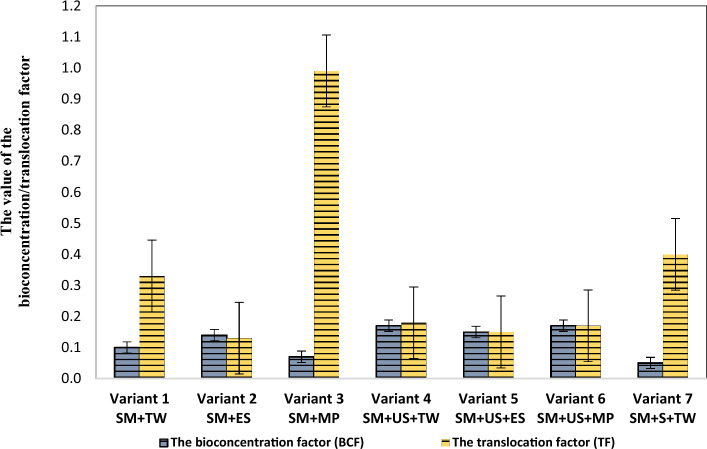
Factors of lead bioconcentration and translocation in *Robinia pseudoacacia* for the investigated experimental variants. *SM* sterile material, *TW* tap water, *ES* enzyme solution, *MP* solution based on KH_2_PO_4_, *US* unpolluted soil, *S* sludge.

**Figure 4:**
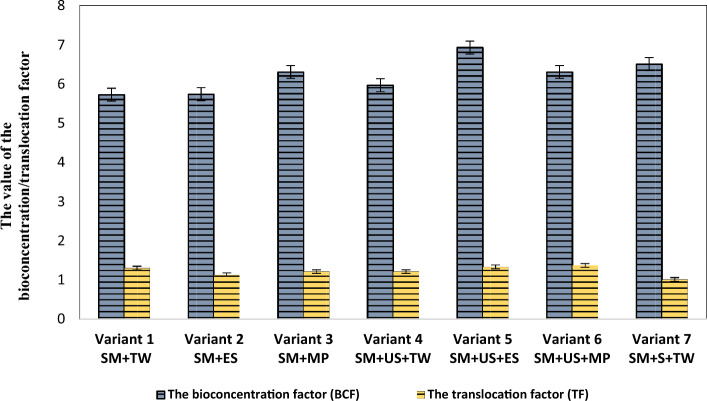
Copper bioconcentration and translocation factors in *Robinia pseudoacacia* for the investigated experimental variants. *SM* sterile material, *TW* tap water, *ES* enzyme solution, *MP* solution based on KH_2_PO_4_, *US* unpolluted soil, *S* sludge.

The bioconcentration (BCF) and translocation factors (TF) were used to further evaluate the ability of *R. pseudoacacia* to accumulate lead and copper from the sterile material and the obtained results are shown in Figs. 3 and 4 in the case of every prepared experimental variant.

As can be seen in Fig. 3, the accumulation and translocation factor in the case of lead showed values ˂ 1 for every investigated experimental variant, indicating that *Robinia pseudoacacia* tends to concentrate lead in the roots and around its roots.

In the case of copper, the results obtained from the point of view of bioconcentration and translocation factors, shown in Fig. 4, indicate that the *Robinia pseudoacacia* is appropriate to extract the copper from the investigated sterile material since both factors had values > 1 for all the experimental variants.

In detail, for experimental variants 4 and 6, in which unpolluted soil was used, the bioconcentration factor of lead showed a value of 0.17, and in the case of experimental variants 1 and 3, in which only sterile material was used the assessed bioconcentration factors were 0.1 and 0.07, respectively. The assessed bioconcentration factors for copper in the investigated experimental variants range from 5.72 to 6.92. As regards the translocation factors for lead, values range from 0.13 to 0.99 while for copper the assessed values range from 1.01 to 1.37.

### The yield of the phytoremediation process using black locust

**Figure 5:**
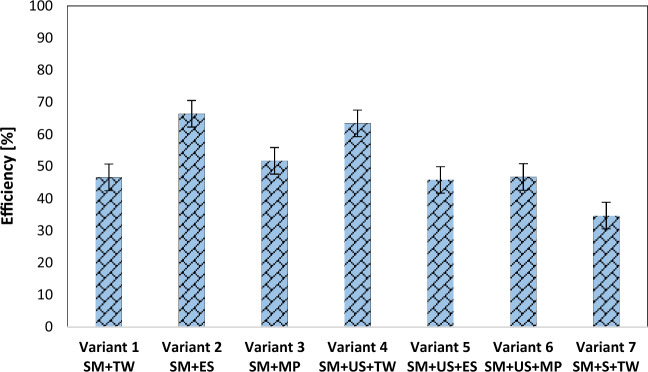
The yield of the lead phytoextraction process from the experimental variants. *SM* sterile material, *TW* tap water, *ES* enzyme solution, *MP* solution based on KH_2_PO_4_, *US* unpolluted soil, *S* sludge.

**Figure 6:**
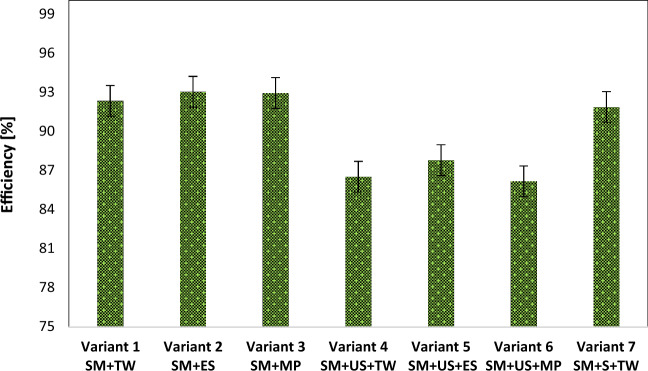
The yield of the copper phytoextraction process from the experimental variants. *SM* sterile material, *TW* tap water, *ES* enzyme solution, *MP* solution based on KH_2_PO_4_, *US* unpolluted soil, *S* sludge.

The yield of the phytoremediation process with *Robinia pseudoacacia*, in the case of the prepared experimental variants, is shown in Figs. 5 and 6, for copper and lead, respectively. The highest yield was reported in the case of copper, especially in the experimental variants which consisted of only sterile material.

Experimental variants 1, 2, and 3 showed copper extraction yield values in the range of 92.3–93.0%. The 93.0% yield was found in variant 2, on which the solution from “Super Enzymes” pills was used. Experimental variants 4, 5, and 6 showed copper extraction yields in the range of 86.1–87.7%. In the composition of experimental variants 4, 5, and 6, 500 g of sterile material and 500 g of unpolluted soil were used. Among these, the highest yield was found in experimental variant 5, on which the solution from “Super Enzymes” pills was also used.

In the case of lead, when the yields of the phytoextraction process from the experimental variants prepared in plants were determined, the values recorded were in the range of 45.8–66.4%. In experimental variants 1, 2, and 3, the yield of the phytoextraction process fell within the range of 46.6–66.4%. The percentage noted in experimental variant 2 was 66.4%. The yields of experimental variants 4, 5, and 6 indicated values ranging from 45.8 to 63.4%. In the experimental variant 4, the yield obtained was 63.4%.

Experimental variant 7, in the composition of which there were 800 g of sterile material and 200 g of dehydrated sludge, reflected a 91.8% yield in the case of copper and a 34.7% yield in the case of lead.

## Discussion

The sterile dumps from mining areas present a limiting environment characterized by low levels of nutrients (K, N, P) and significant amounts of heavy metals (e.g. Cu, Pb, Zn, Ni, Cd, etc.) plus a poor structure which displays a strong pressure on flora requesting more adaptive and resistant plants. The high metal content assessed in sterile material taken from the “Radeș” sterile dump confirmed the extreme metal pollution of this mining area. It has been reported that heavy metals have a toxic effect on plants (e.g. Pb, Cd, As, Cr) because they interfere with nutrient transport and absorption and some of them are essential for plant metabolism (photosynthesis, respiration, enzyme activity, gene regulation, sugar metabolism, nitrogen fixation) but at low concentrations (e.g. Cu, Ni, Zn, Fe, Mn, Mo)^24,29^. For example, copper is the component of many enzymes and proteins engaged in the processes of respiration and photosynthesis, but in excess, it may cause chlorosis, root growth suppression, and damage the permeability of cell membrane^30^. When heavy metals in greater quantities enter plants' systems through their roots and leaves, interferences with their physiological and metabolic functions as well as plant growth and development issues appear^29,31^. The homeostasis and ion distribution in plant cells is perturbed leading to an enhancement in the accumulation of reactive oxygen species. The ability of plants to scavenge the toxic effects of reactive oxygen species seems to be the most important determinant of their tolerance. To overcome stress conditions related to the presence of huge quantities of heavy metals, the tolerance mechanism of plants includes some physio-biochemical strategies in a complex antioxidant defence system which includes numerous enzymatic components (e.g. superoxide dismutase, catalase, peroxidases, glutathione peroxidase, glutathione reductase, ascorbate peroxidase) and non-enzymatic components (e.g. ascorbic acid, glutathione, phenolic compounds, alkaloids, flavonoids, carotenoids, free amino acids, proline). These enzymes protect various components of the cells from damage and play an important role in plant growth and development by modulating cellular processes^32^.

Despite these unfavorable conditions for plant growth, previous studies confirmed that some plant populations survive in solid wastes from mining areas. Thus, *Lupinus an-gustifolius, Helichrysum microphyllum* subsp. *Tyrrhenicum, Solanum viarum Dunal, Quercus* spp. and* Salix* spp. species were successfully used for phytoremediation of mining tailings and mining dumps contaminated with 46.89–259.7 mg kg^−1^ Cu, 2–6090 mg kg^−1^ Pb, 0.9–2975 mg kg^−1^ Zn, 15.3–32.42 mg kg^−1^ Cr, and 3.24–65.0 mg kg^−1^ Cd^20–22,24,27^. Also, *Robinia pseudoacacia, Pinus elliottii Engelmann,* and *Populus tomentosa Carr.* have demonstrated abilities to adapt to high-density sludge sediment of copper mines^23^. The *Robinia pseudoacacia* in this experiment was able to survive in sterile material contaminated with important quantities of Cu (424 mg kg^−1^) and Pb (2489 mg kg^−1^).In the case of all prepared experimental variants, the lead and copper concentration found in the bulk material and the material from the rhizosphere of *black locust* at the end of the experiment showed a decrease compared to the initial results. As regards lead, the alert threshold provided in the Romanian legislation^33^ for less sensitive soils (250 mg kg^−1^) was exceeded for all experimental variants while, in the case of variants 1, 3, and 7 the intervention threshold was exceeded (1000 mg kg^−1^). Concerning copper, the recorded values were lower than 33 mg kg^−1^ and the alert (250 mg kg^−1^) and intervention (500 mg kg^−1^) thresholds provided in Romanian legislation for less sensitive soils were not exceeded.

In the present study, the accumulation of lead and copper in the material from the rhizosphere of *black locust* depends on the content of the investigated experimental variants. A maximum of lead accumulation in the material from the rhizosphere of *black locust* was recorded in the experimental variant which contained only sterile material while a minimum of accumulation was observed when sterile material was mixed with unpolluted soil. This discrepancy may be attributed to the strong interaction of lead with organic matter present in the unpolluted soil which causes poor mobility and immobilization of lead within the soil matrix^34^.

Despite the high content observed in the bulk material and the material from the rhizosphere of *black locust*, the roots of the *Robinia pseudoacacia* developed in investigated variants accumulated up to 904.0 mg kg^−1^ of lead. In contrast, in the aerial parts, the values did not exceed 140.96 mg kg^−1^ of lead. The lead concentrations in *Robinia pseudoacacia* tissues were similar to those of other species^21^. Among the tested experimental variants, the highest values of lead in roots were recorded when sterile material was irrigated with enzyme solution, indicating a favorable effect on lead uptake by roots. This positive effect may be due to the contribution of enzymes to ensure necessary nutrients for plants and alleviate the plants’ stress conditions. Antioxidant enzymes are the first line of defence against the damages caused by free radicals, are critical for the optimum health of plant cells and play a significant role in assisting plant development by modulating cellular processes such as mitosis and a wide range of processes such as cell growth/division, regulation of senescence and sulphate transport, detoxification, synthesis of proteins and nucleotides, and expression of stress responsive genes^32^. In this case, our results regarding lead uptake by the roots of *R. pseudoacacia* are better than the ones reported for other species^21^.

Among the investigated variants, the differences between the concentration of Pb in *R. pseudoacacia* roots and the ones observed in the material from the rhizosphere of *black locust* may be related to a very low concentration of bioavailable Pb that was present in the substrates indicating that only a little amount of lead is available for the plant’s roots as suggested by another study which investigated the ex-situ phytoremediation of a mine waste using pioneer species^24^.

The results obtained within this study indicate that in the dry biomass of *Robinia pseudoacacia*, the concentration of Pb was concentrated in the roots compared to the above-ground parts for all the investigated variants, which could be related to the low mobility of this element in the plant. Hence, *Robinia pseudoacacia* can uptake only a little concentration of lead into the epigean organs in accordance with the results reported in the case of soil phytoremediation using frugal *Helichrysum italicum (Roth) Don*. which accumulated greater amounts of heavy metals, especially Cr, Cu, Pb and Zn in their root system^35^. Other studies corroborate with the present study since the authors also found Pb concentration higher in the roots of *Helichrysum* microphyllum *subsp. Tyrrhenicum*^24^. In a previous study carried out by Kahle^36^, it was suggested that a high accumulation of metals in the roots is related to the immobilization of these metals by insoluble organic polymers present in the plant tissue.

In addition, a potential presence of an exclusion system for Pb may be suggested, or a reduced Pb bioavailability. Even if is not an essential element for plant nutrition, when Pb is bioavailable in the soil, it can be absorbed by roots and then translocated to aerial parts of the plants or can be deposited in roots as exclusion mechanism. In the case of other species, it was found that Pb is deposited in the cell walls of the root tissues mainly as pyrophosphate and a certain protective function in the roots can be performed which impedes the movement of lead through the intercellular space restricting it transition into conducting tissues^37^. Other studies identified an exclusion system of *Helichrysum tyrrhenicum* for Zn since this metal was mainly present in the epidermis of *Helichrysum tyrrhenicum* roots^38^ and a poor Pb bioavailability^39^. Nevertheless, the amount of Pb accumulated in *Robinia pseudoacacia* leaves was in the range of 69–140 mg kg^−1^, while other researchers reported ranges as follows: 7.5–39 mg kg^−1^^40^; 30.7 mg kg^−1^^19^; 21.66 mg kg^−1^^15^; 6–15.5 mg kg^−1^^41^; 2.7–4.12 mg kg^−1^^42^.

In the case of copper, an increase in concentrations was found in the aerial part of *black locust* (stems + leaves) and in the roots for all the investigated experimental variants with slight variations among the investigated variants and plant tissues. This suggests that copper has elevated mobility in the plant and preferentially accumulates in the aerial parts of *black locust*. These findings did not agree with those of Muro-González et al.^43^, in which an increase in Cu bioaccumulation was observed in the aerial tissues of *P. laevigata* growing on mine tailing substrate from Huautla, Morelos, while Cu bioconcentration was reduced in the roots of these plants. However, this finding is in accordance with other studies as regards the Cu accumulation in roots in the case of apple trees and *Vitis vinifera* ssp. *syl-vestris*^44,45^ and contrary to the results obtained by Brun et al.^46^ as regards the Cu accumulation in aerial parts in the case of *maise*. Another study in which high values of Cu were identified in two *Tunisian varieties of bean (Mamdouh)* and *faba bean (Badii)* suggested that Cu is readily translocated to the aerial parts of the plants, owing to its role in plant metabolism because after all, it is a necessary micronutrient^47^. Moreover, the high bioavailability of Cu is possibly due to its high solubility under acidic conditions as free water ions as reported in the case of Cd^48^.

The healthy growth of plants requires small amounts of Cu (20–30 mg kg^−1^) and Pb (5–10 mg kg^−1^) as reported in the literature^20^ and recognized as being sufficient or normal values. The mean heavy metals identified in the dry biomass of *Robinia pseudoacacia* were above the toxicity limits reported in the literature for Cu (20–100 mg kg^−1^) and Pb (30–300 mg kg^−1^)^20^. However, during the performed experiment no development issues were noticed on *Robinia pseudoacacia* plants, which indicates that the development of *Robinia pseudoacacia* plants was normal. Our previous study indicated the development of *Robinia pseudoacacia* plants in sterile material^49^.

There are two coefficients worth mentioning, namely the bioaccumulation factor and translocation factor, that allow judging the suitability of a plant for phytoremediation. If the bioaccumulation factor is higher than one, it means that the plant is a hyperaccumulator while if it is less than 1 the plant can only absorb but not accumulate metal. A translocation factor higher than 1 indicates that the plant translocated heavy metals effectively from root to shoot. However, a translocation factor smaller than 1 indicates ineffective metal transfer suggesting that these types of plants accumulate metals in the roots more than in shoots or the leaves. In hyperaccumulators, both ratios should be greater than 1. Plants with a high bioaccumulation factor in aboveground parts of plants are suitable for phytoextraction while plants with a high bioaccumulation factor for belowground parts of plants combined with a translocation factor less than 1 are suitable for phytostabilization^16^.

In the present study, the ability of *Robinia pseudoacacia* to accumulate lead and copper from sterile material was evaluated by the bioconcentration and translocation factors. Therefore, the bioconcentration and translocation factor in the case of lead showed values ˂ 1, which results that the *Robinia pseudoacacia* is suitable for phytostabilization of tailings dumps with high lead content, accumulating high concentrations around the roots and in the roots. In the case of copper, both bioconcentration and translocation factors show values greater than one, which indicates that *Robinia pseudoacacia* is appropriate for extracting high concentrations of copper from the sterile material and therefore the official criteria^16^ that allows its classification as a hyperaccumulator for Cu have been met.

Regarding the translocation factors and the bioaccumulation factor, there is a lack of specific literature on *black locust*. The assessed bioaccumulation factor values for lead (< 1) are consistent with the ones reported in the case of other species such as *D. viscosa subsp. viscosa, E. pithyusa* subsp.* cupanii, Helichrysum microphyllum* subsp.* tyrrhenicum*, and *C. salviifolius*^24,50^.

The values of translocation factors for Cu in *Prosopis laevigata* (> 1)^32^ and *Solanum viarum Dunal*^20^ were similar to the values found in this study. According to Kabata-Pendias and Dudka^51^, the accumulation efficiency can be intensive (BAF > 1), medium (BAF = 1–0.1), and weak (BAF = 0.1–0.01). Therefore, the Cu accumulation efficiency of *Robinia pseudoacacia* may be regarded as intensive. Concerning the bioaccumulation factors observed for copper using *Robinia pseudoacacia* the maximum value obtained in the case of Cu in the leaves of *S. caprea* (8.54)^52^ was higher than the maximum value among our results (6.92).

The results of our study revealed that *Robinia pseudoacacia* may be a possible efficient candidate for phytoremediation and treatment of sterile dumps contaminated with copper and lead. A comparative analysis of the phytoremediation capabilities of *Robinia pseudoacacia* with those formerly reported for other plants used for the phytoremediation of different mining waste sites is summarized in Table 1.

**Table 1:** Comparison of the phytoremediation capabilities for Cu and Pb of various plants used for the phytoremediation of different mining waste sites.

Plant	Medium of application	Metal concentration (mg kg^−1^)			Phytoremediation indexes		Refs.
		Rhizosphere	Roots	Shoots	BCF	TF	
*Solanum viarum*	Copper mining tailing	–	229 (Cu) 33 (Pb)	366 (Cu) 42 (Pb)	1 (Cu) 16.4 (Pb)	1.3 (Cu) 1 (Pb)	^20^
*Robinia pseudoacacia (black locust)*	Sludge sediment of copper mine	–	148.78 (Cu) 4.99 (Pb)	37.96* (Cu) 2.09* (Pb)	0.359 (Cu) 0.321 (Pb)	1.571 (Cu) 1.293 (Pb)	^23^
*Pinus elliottii Engelmann*		–	39.53 (Cu) 0.99 (Pb)	19.87* (Cu) 3.99* (Pb)	0.070 (Cu) 0.093 (Pb)	2.086 (Cu) 16.75 (Pb)	
*Populus tomentosa Carr*		–	86.12 (Cu) 1.88 (Pb)	20.35* (Cu) 4.25* (Pb)	0.145 (Cu) 0.143 (Pb)	0.510 (Cu) 3.608 (Pb)	
*Helichrysum microphyllum subsp. tyrrhenicum*	Mine waste (sterile material)	5030 (Pb)	680 (Pb)	1020 (Pb)	0.14 (Pb)	1.50 (Pb)	^38^
*Robinia pseudoacacia (black locust)*	Sterile material	27.9 (Cu) 1543.7 (Pb)	154 (Cu) 904 (Pb)	189 (Cu) 140.96 (Pb)	6.3 (Cu) 0.14 (Pb)	0.99 (Pb) 1.3 (Cu)	This study

*Value recorded in leaves.

As shown in Table 1, the performances of *Robinia pseudoacacia* in the phytoremediation of sterile material were found comparable and competitive with those reported for other plants previously used for phytoremediation of mining waste sites. The translocation and the bioaccumulation factors of the *Robinia pseudoacacia* are also similar to the values reported in the literature.

From the obtained results it was found that the experimental variants prepared from 1000 and 800 g of sterile material, without being mixed with unpolluted soil (the case of variants 1, 2, 3, and 7) reflected higher yields both in the case of the extraction of lead (46.64–66.41%) and copper (92.3–93%). The maximum yield of the phytoextraction process obtained both in the case of copper and lead was found in experimental variant 2 which contained only sterile material and on which a solution from “Super Enzymes” pills was used as a watering solution. Other studies reported the benefit of a proper chemical agent application that increases phytoavailability and uptake of metals together with a reduction of their phytotoxicity and consequently an increase in phytoremediation efficiency^53,54^. That opens the possibility of further research in applying enzymatic solutions on *R. pseudoacacia* and other plant species for increasing the phytoremediation efficiency of sterile dumps.

## Future perspectives

Although the promising results obtained within this preliminary work offer an ecological method to reduce the environmental impact of heavy metals and increase phytoavailability and uptake of Cu and Pb by *Robinia pseudoacacia* through enzyme-mediated phytoremediation together with a reduction of their phytotoxicity, further research, including practical field-scale application for sustainable sterile dumps rehabilitation is needed.

To achieve this, long-term lab-scale investigations are necessary to assess the effectiveness of this approach and to optimize the phytoremediation process. Further lab-scale investigation may be related to optimizing the dose of enzymatic solution and application methods to improve phytoextraction efficiency. Additionally, studying the interactions between *Robinia pseudoacacia* and sterile material (irrigated with enzymatic solution) at the molecular and microbial level can provide insights into enhancing metal uptake pathways, thus improving (enzyme-mediated) phytoremediation efficacy. Furthermore, the adverse effects of enzymatic solution on the environment (especially soil, surface, and groundwater) and on plant vitality over time must be thoroughly investigated.

Under real-world conditions, the feasibility and effectiveness of enzyme-mediated phytoremediation of sterile dumps using *Robinia pseudoacacia* plant should also be validated by using a combination of novel techniques, such as nanoparticles and biochar, in the laboratory to field-scale experiments.

Nanotechnology has many uses in agriculture, industry, medicine, and environmental protection and resulted in a revolutionary improvement in plant growth and protection^55,56^. Nanofertilizers like bio-fertilizer, major elements nanofertilizers like nano-nitrogen chelate, and nano-vermicompost organic fertilizer could improve nutrient delivery to plants because have greater uptake efficiency and fewer adverse environmental effects. Likewise, nanoparticle seed coating has shown its efficiency in increasing germination rates and providing early defense against environmental hazards. The mechanism of the accumulation of nanoparticles in the cell wall of plants is still not fully explored^57^ and increasing the distribution of nanomaterials in different field experiments is also crucial (including studies on sterile material).

Furthermore, phyto-nanotechnology is one of the most applicable methods for remediating or detoxifying hazardous pollutants by decreasing reactive oxygen species production and restricting heavy metal release. For example, extreme reactive oxygen species caused by cadmium stress were reduced when Si-nanoparticles were applied to rice seedlings^58^. Also, plants exposed to nanoparticles create more antioxidant molecules, both enzymatic (superoxide dismutase, catalase) and non-enzymatic (ascorbate, glutathione, carotenoids, tocopherols, and phenolics), that are linked to plant defense systems^55,59^. Further future perspectives towards the studied problem of removing Cu and Pb from sterile material using *Robinia pseudoacacia* plant through enzyme-mediated phytoremediation could be applying phyto-nanotechnology to increase the expression of used antioxidant enzymes. In this regard, it was reported that chitosan-polyvinyl alcohol hydrogel with absorbed copper nanoparticles can increase the lycopene content and total antioxidant capacity in tomato fruits^60^.

The combination of biochar and nanoparticles can help mitigate plants' abiotic stress caused by heavy metals. Biochar acts as a nutrient reservoir and facilitates nutrient cycling and has long been recognized for its capacity to adsorb heavy metals, thus preventing their uptake by plants. The integration of biochar with nanoparticles maintains adequate nutrient levels for plants even under stressful conditions caused by heavy metals. However, the effectiveness of the combination of biochar and nanoparticles in addressing plant stress may vary based on the specific stress factor, plant species, and soil type^55^. In this regard, studying the effect of biochar and nanoparticle combinations in addressing *Robinia pseudoacacia* stress when exposed to sterile material could be important.

Moreover, the influence of heavy metal-tolerant bacteria, biochar, and their joint application on *Hordeum vulgare* L., grown in highly polluted soils was investigated recently. The metal-tolerant bacteria + biochar improved the growth performance of plants in polluted soil, leaves lengths reaching those of unpolluted control^61^. Related to the results of the present research, investigating the influence of metal-tolerant bacteria in enzyme-mediated phytoremediation of sterile dumps using *Robinia pseudoacacia* plant may be valuable.

Despite remarkable progress in using nanotechnology administering nanoparticles, exogenous enzymes, bacteria, microbes and combinations with other materials or techniques remains a complex task. To demonstrate that these nanomaterials, exogenous enzymes, or other substances are helpful to plant growth and do not have adverse effects on animal or human health, prolonged experimental observations are required.

## Materials and methods

### Study area

**Figure 7:**
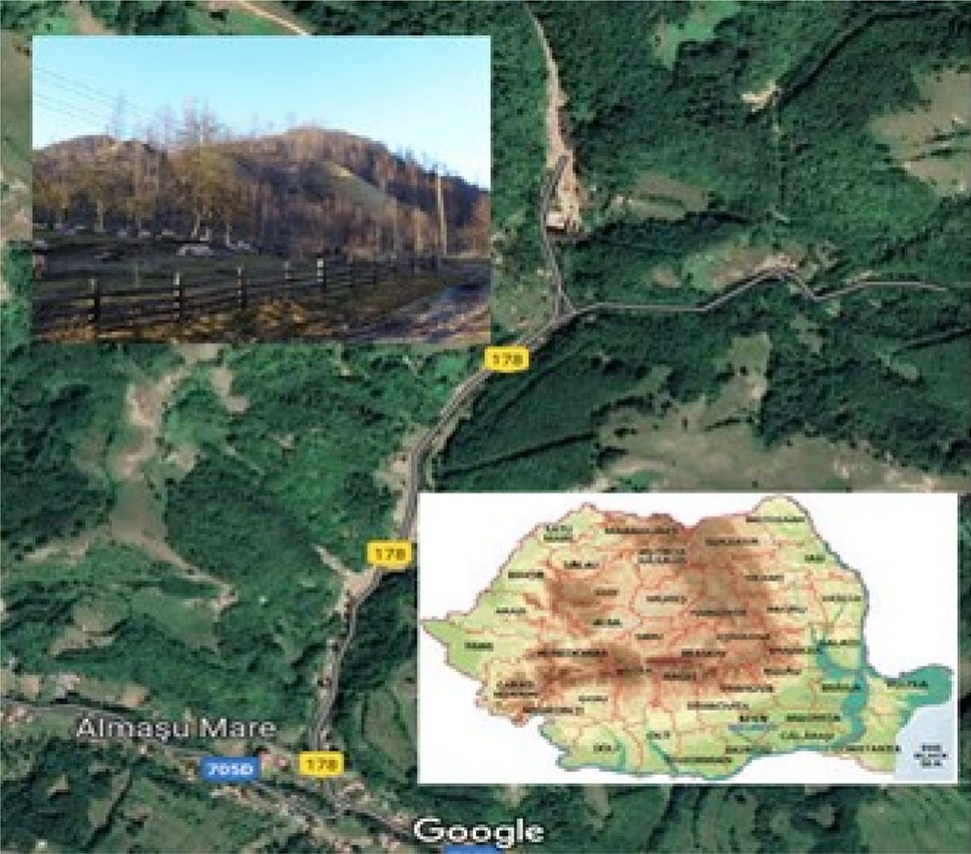
The geographical location of the “Radeș” dump in Alba County in Romania.

The “Radeș” exploitation (GPS coordinates: 46° 07014.700 N, 23° 07027.200 E), the subject researched in this paper, is in the Almașu Mare commune of Alba County (Romania) (Fig. 7), on the southern outskirts of the Apuseni Mountains and the Metaliferi Mountains subunit. This area is part of a mining field located in the southeast of the Zlatna-Stănija volcanic alignment (Romania)^49^.

The study area is characterized by a unique historical and industrial heritage of the exploitation of gold in Romania. Since 1900, mining activities have been performed in this area. Today, the mining activity has been completely stopped at the “Radeș” exploitation. However, the area of the “Radeș” mine is highly polluted with heavy metals due to the improper storage of the gangue material extracted from the “Radeș” gallery, which allowed an unimpeded flow of acid mine drainage. The dump composed of gangue material extends over a distance of about 100 m, the width is 20 m, the high is about 30 m, and is located approximately 50 m from the “Radeș” mine gallery (hence the name of the "Radeș" dump). The stored waste material represents the unusable part of the ore deposit, the part that was removed at the end of the ore preparation technological process^62^) and is characterized by its strong acidic nature and by significant amounts of lead, copper, and other heavy metals^63^. Heavy metals have accumulated in the soil below the submerged dump, but also in the neighboring soils due to road transport, the aerial movement of dust from the surface of the dump (through the wind), and the distribution of underground and surface water, thus creating large imbalances in the soil ecosystem and the entire local ecosystem. Human houses and outbuildings are very close to this source of pollution that poses a significant risk to the quality of the environment and human health.

To analyze the current situation of the “Radeș” sterile dump, samples of gangue material were taken to determine the concentrations of heavy metals. The results obtained showed that the studied area is polluted with heavy metals, especially lead (2489 mg kg^−1^) and copper (424 mg kg^−1^), which represents a significant risk for the existing residents of the area. In the case of Cu, the concentration exceeds the alert threshold stipulated in the legislation^33^, and the Pb concentration exceeds twice the intervention threshold and over 10 times the alert threshold for less sensitive land use.

Following the study of the current situation of the study area, an experimental model of phytoremediation with *Robinia pseudoacacia* was created, at the laboratory level, to identify an optimal solution to remedy the current situation of the abandoned sterile dumps. The experimental phytoremediation model involved 4 stages, namely the preparation of the experimental variants, the sowing of *Robinia pseudoacacia* seeds in the prepared experimental variants, the preparation of spraying solutions, the determination of the Pb and Cu concentration in the experimental variants, and the tissues of the *black locust*. Based on the results obtained the bioconcentration and translocation factors, and the yield of the phytoremediation process were assessed. After the phytoremediation experiment which lasted 12 weeks, samples were taken from sterile material corresponding to every prepared experimental variant, from the material from the rhizosphere of *black locust*, and from the tissues of the harvested plants. Each experimental variant prepared was homogenized, multiplied three times, and analyzed using the SHIMADZU AA-6800 atomic absorption spectrophotometer, three times, reporting the average value.

### Preparation of experimental variants

The preparation of the experimental variants consisted of the mixing of several types of homogeneous materials placed in polypropylene pots, with a diameter of 23 cm and consisting of the following: sterile material taken from the “Radeș” dump, soil collected from a non-industrialized mountain area (Cotorăști village, Râmeț commune, Alba county) and dewatered sludge resulting from the Someș sewage treatment plant in Cluj Napoca according to Table 2. Also, part of the prepared variants was watered once a week with potassium monobasic phosphate (KH_2_PO_4_—99.5%) and others with enzyme solution from “Super Enzymes” tablets taken from the market (Table 2). Each experimental variant was multiplied three times.

**Table 2:** The composition of the pots used in the phytoremediation experiment.

Experimental variant	Composition and watering/irrigation solution	Variants codes
Variant 0	Unpolluted soil (reference) (1000 g) + tap water	US + TW
Variant 1	Sterile material (1000 g) + tap water	SM + TW
Variant 2	Sterile material (1000 g) + enzyme solution	SM + ES
Variant 3	Sterile material (1000 g) + solution based on KH_2_PO_4_ (99.5%)	SM + MP
Variant 4	Sterile material (500 g) + unpolluted soil (500 g) + tap water	SM + US + TW
Variant 5	Sterile material (500 g) + unpolluted soil (500 g) + enzyme solution	SM + US + ES
Variant 6	Sterile material (500 g) + unpolluted soil (500 g) + solution based on KH_2_PO_4_ (99.5%)	SM + US + MP
Variant 7	Sterile material (800 g) covered with sludge (200 g) + tap water	SM + S + TW

The sterile material was harvested from the “Radeș” tailings dump with the help of a manual drill, from a depth of 10–30 cm.

The soil collected from the Cotorăști area was used as a reference soil and was sampled in the same way as the sterile material. The reference soil was used to prepare experimental variant 0, which was chosen as the control variant.

Generally, the dewatered sludge resulting from urban sewage treatment plants is composed of 20% fats, 50% carbohydrates (sugar, starch, and fiber), 30–40% organic matter, 3% total nitrogen, 1.5% total phosphorus, and 0.7% total potassium, has a C:N ratio of 10–20 and a neutral pH and it is often used as an organic fertilizer for acidic soils improving the soil physico-chemical properties, being an ecological alternative to chemical fertilizers^26,64^. The dewatered sludge resulting from the Someș sewage treatment plant did not record traces of heavy metals.

### Sowing of *Robinia pseudoacacia* seeds in experimental variants

*Black locust* is considered to be suitable for planting on heavy metal-polluted soils due to its rapid growth, deep root system, high tolerance to heavy metal concentrations, and ability to fix heavy metals around the roots^65–67^. *Black locust* can be successfully used to stabilize slopes, against landslides, stabilizing sand dunes, or even abandoned tailings dumps^68^. Given these qualities of *Robinia pseudoacacia*, it was used in the phytoremediation experiment implemented at the laboratory level, for 12 weeks. Experimental phytoremediation lasted 12 weeks, from the sowing of the seeds in the pots to the harvesting of the plants.

The seed preparation activity consisted of the next treatment: approximately 500 seeds were placed in a plastic container over which heated tap water was poured. The seeds were left in water for 24 h after which they were introduced into the mixtures prepared in pots (experimental variants). The purpose of heating the water and pouring it over the seeds was to soften their shells so that when the germination process begins the seedlings inside the material can easily break apart and come to the surface. After the preparation of the experimental variants, 10 *Robinia pseudoacacia* seeds were introduced into each pot, at a depth of 2–3 cm. Before sowing, each pot was immersed in a bucket of tap water so that the composition had the necessary moisture for seed germination. In order to optimally carry out the phytoremediation experiment with *Robinia pseudoacacia*, at the laboratory level, the necessary conditions for the development of the plants were ensured, such as: natural light (15 h of light and 9 h of darkness, each day with a 24-h cycle), humidity (30–50%), and temperature (22 ± 2 °C). The seeds of *Robinia pseudoacacia* used in this study came from commercial plants and were provided by Ferma Bârzani S.R.L.

### Preparation of spraying solutions

The pots with the prepared experimental variants were watered once a week with potassium monobasic phosphate KH_2_PO_4_ (99.5% concentration) and with enzyme solution from “Super Enzymes” tablets taken from the market, as can be seen in Table 2. The enzyme tablets contain 100% natural ingredients. When preparing the solution with “Super Enzymes” tablets, two pills from the box were crushed and mixed with 350 mL of tap water, and then 50 mL of the solution was applied to the corresponding pots. This enzymatic treatment was applied to dispose of the mineral ions and facilitate their absorption. The enzymatic preparation contains cellulases that can break down cellulose from the soil, along with proteases, lipases, and glycolytic enzymes that also break down organic material and make mineral ions available.

The preparation of the monobasic potassium phosphate solution consisted of weighing 0.05 g of KH_2_PO_4_ which was diluted with 350 mL of tap water. As in the case of the enzyme solution, 50 mL was taken from the solution prepared with monobasic potassium phosphate and applied to the corresponding pots.

### Determination of the Pb and Cu concentration in the experimental variants

The determination of the concentrations of lead and copper in the experimental variants was carried out using a method compliant with SR ISO 11466:1999 regarding the extraction of microelements from the soil in aqua regia for the determination of the total form^69^. From the dry and ground sample of each experimental variant, 3 g were taken, then 1 mL of distilled water, 21 mL of concentrated HCl (32%), and 7 mL of concentrated HNO_3_ (65%) were added.

After mineralization, the obtained samples were filtered, and then the concentrations of lead and copper were determined using the SHIMADZU AA-6800 atomic absorption spectrophotometer.

### Determination of the Pb and Cu concentration in the plant parts of black locust

**Figure 8:**
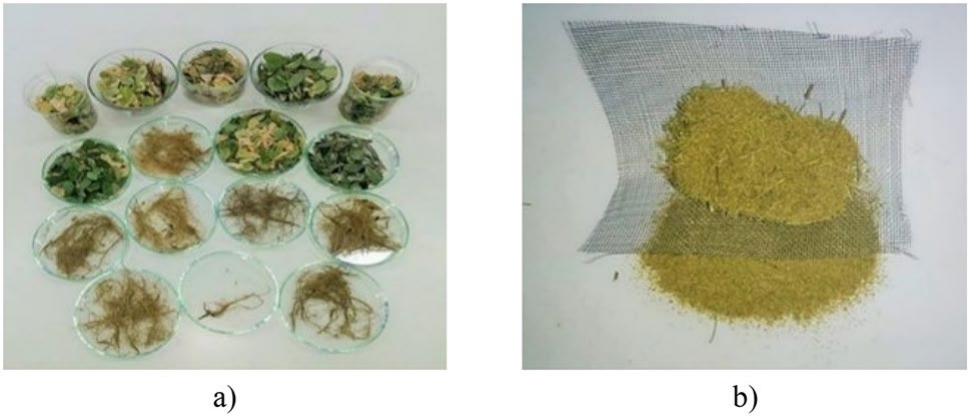
Preparation of plant samples for drying (**a**) and mineralization (**b**).

To assess the capacity of the *Robinia pseudoacacia* absorption of lead and copper concentrations from the prepared variants, after the 12 weeks of plant growth, they were harvested, and subsequently, samples were taken from the roots and aerial parts of the plant (stems and leaves). The roots of *black locust* grown in each type of prepared experimental variant were dried in an oven at approximately 105 °C and then ground using laboratory mills (Fig. 8). From each subsample, 0.5 g was weighed, with an accuracy of 0.001 in a Berzelius beaker, covering it with a watch glass after adding, under stirring, 2 mL of 30% hydrogen peroxide (perhydrol).

The dried roots in the Berzelius beaker were allowed to react with the perhydrol for about 3 h. After this time, 6 mL of concentrated nitric acid was added drop by drop, and then they were placed on the sand bath and left to mineralize for about 2 h.

The insoluble residue in the reaction beaker was allowed to settle. The supernatant obtained by decantation was passed in a 100 mL flask through filter paper, which was then brought to the level with distilled water. From the solutions obtained, the concentrations of lead and copper were determined by atomic absorption spectrophotometry using the SHIMADZU AA-6800 spectrophotometer. Regarding the multiplication, the same was done as in the case of the soil samples, the average results being reported. In this study, the comparison between treatments and comparisons between treatments and/or between roots/aerial parts for copper and lead was followed. To identify the potential of the *Robinia pseudoacacia* for phytostabilization or phytoextraction, the metal fraction adsorbed or surrounding the root (adsorbed) in the rhizosphere was determined. These concentrations were determined as described in “Determination of the Pb and Cu concentration in the experimental variants” section (for soil), respectively this section (for plant parts).

The testing of the parameters was determined by 3 identical, consecutive measurements, to ensure the conditions of repeatability and reproducibility, the reported value represented the arithmetic mean of the experimental results. For the analysis of the degree of precision, based on the determined individual values, the mean square deviation and the type A measurement uncertainty were calculated.

The limit of detection (LOD) was calculated as the ratio between 3 times the standard deviation resulting from 10 measurements of the reagent blank and the slope of the calibration curve (Table 3).

**Table 3:** The limit of detection (LOD) of the metals investigated.

Parameter	Pb (µg L^−1^)	Cu (µg L^−1^)
LOD	0.270	0.200

For the quality of the results, calibration standards, and procedural blank measurements were used. Multielement standard solution (ICP multielement Merck, Darmstadt, Germany) was used in the case of the spectrometer calibration. The accuracy of the heavy metals determination was tested by analyzing the metal concentration of the European Reference Material (ERM-CC018) Standard concentrations (BAM Division I.1, Berlin, Germany). The mean ranged between 97 and 104%. During all experiments and studies, all rules and regulations were followed by the authors. The formal identification of the plant material used in our study was undertaken by S.L. Dr. Eng. Budău Ruben of the University of Oradea. No voucher specimen of plant material has been deposited in a publicly available herbarium.

### Determination of bioconcentration and translocation factors

The bioconcentration factor (BCF) is defined as the total concentration of the elements in the plant tissue/the total concentration of the elements in the sterile material^70,71^. Bioconcentration of lead from plant tissue and soil can be calculated using the following equation^72^:

1 $${\text{BCF}} = {{{\text{C}}_{{\text{plant tissue}}} } \mathord{\left/ {\vphantom {{{\text{C}}_{{\text{plant tissue}}} } {{\text{C}}_{{{\text{soil}}}} }}} \right. \kern-0pt} {{\text{C}}_{{{\text{soil}}}} }}$$

where C_plant tissue_ and C_soil_ are the concentration of metals in the plant layer (mg kg^−1^) and the soil (mg kg^−1^), respectively. BCF was further classified as hyperaccumulators, accumulators, and exclusions in samples that accumulated metals > 1 mg kg^−1^ and < 1, respectively^72–74^. For a plant to be considered a good accumulator, the value of the bioconcentration factor must exceed the value of 1. The bioconcentration factor of a species varies in a ladder range depending on heavy metal concentrations, the type of soil texture, pH, matter content organic, etc.) and climate^71^.

The translocation factor (TF) or plant/root tissue ratio is defined as the total element concentration in plant tissue/total element concentration in root tissue^71^. The translocation of lead from the root to the aerial part can be determined using the following equation^72^:

2 $${\text{TF}} = {{{\text{C}}_{{\text{plant tissue}}} } \mathord{\left/ {\vphantom {{{\text{C}}_{{\text{plant tissue}}} } {{\text{C}}_{{{\text{roots}}}} }}} \right. \kern-0pt} {{\text{C}}_{{{\text{roots}}}} }}$$

where C_plant tissue_ and C_*roots*_ represent the metal concentration in the plant part (aerial) (mg kg^−1^) and the root (mg kg^−1^). TF > 1 represents the fact that the translocation of metals took place in stems and leaves from the root^72,73,75–77^.

Plants with both factors (translocation factor and bioconcentration factor) > 1 are suitable for phytoextraction, while plants with both factors < 1 are suitable for phytostabilization^70^.

### Determination of the yield of the phytoremediation process

The final remediation yield for each prepared experimental variant was calculated using the following equation^78,79^:

3 $$\upeta = \frac{{m_{e} }}{{m_{i} }} \times 100 \left( \% \right)$$

where m_e_ represents the metal concentration extracted from the prepared experimental variants (mg kg^−1^); m_i_ represents the initial concentration of the metal in the prepared experimental variants (mg kg^−1^).

### Research involving plants statement

Experimental research and field studies on plants (either cultivated or wild), including the collection of plant material, comply with relevant institutional, national, and international guidelines and legislation.

## Conclusions

The potential of the *Robinia pseudoacacia* in the process of phytoremediation of the abandoned sterile dump "Radeș" from Almașu Mare Commune (Alba, Romania) was investigated through the implementation, at the laboratory level, of a phytoremediation experiment based on various variants prepared from mixtures of gangue material, uncontaminated soil, and dehydrated sludge.

The following important conclusions could be drawn:

1. The *Robinia pseudoacacia* may be an efficient candidate for phytoremediation and treatment of the “Radeș” sterile dump being able to survive in the sterile material contaminated with important quantities of lead (2489 mg kg^−1^) and copper (424 mg kg^−1^).
2. *Robinia pseudoacacia* accumulates greater amounts of lead in its root system (90.42–904 mg kg^−1^) but can uptake only a little concentration of lead into the epigean organs (69.14–140.96 mg kg^−1^) suggesting a potential presence of an exclusion system for Pb, and strategies of phytostabilization.
3. *Robinia pseudoacacia* is appropriate to extract high concentrations of copper from the sterile material, which allows its classification as a hyperaccumulator for Cu since substantial amounts of copper were accumulated in the roots (133.3–189.6 mg kg^−1^) and aerial parts of the plant (172–189 mg kg^−1^).
4. The assessed bioconcentration and translocation factors confirmed the ability of *Robinia pseudoacacia* to remediate a lead and copper-contaminated site through phytoextraction/phytostabilization.
5. The experimental variants prepared only from sterile material, without being mixed with unpolluted soil reflected yields located in the range of 46.6–66.4% for lead and 92.3–93% for copper.
6. A favorable influence in terms of the yield of the phytoextraction/phytostabilization both in the case of copper and lead was observed when gangue material was not mixed with other materials and wetted with the enzymatic solution. In this case, an increase in phytoavailability and uptake of copper and lead by *Robinia pseudoacacia* through enzyme-mediated phytoremediation was noted.

Although the encouraging results obtained within this work offer an efficient method for enzyme-mediated phytoremediation of lead and copper-contaminated sites, further research is needed to optimize the process for sustainable sterile dumps rehabilitation, and understand the mechanisms and processes that take place in the relationship between plant, sterile material, enzyme, and microorganisms.

## Data Availability

The datasets generated during and/or analyzed during the current study are available from the corresponding authors on a reasonable request.
